# The intrinsic circadian clock in podocytes controls glomerular filtration rate

**DOI:** 10.1038/s41598-019-52682-9

**Published:** 2019-11-06

**Authors:** Camille Ansermet, Gabriel Centeno, Svetlana Nikolaeva, Marc P. Maillard, Sylvain Pradervand, Dmitri Firsov

**Affiliations:** 1 Department of Pharmacology and Toxicology, University of Lausanne, Lausanne, Switzerland; 2 Institute of Evolutionary Physiology and Biochemistry, Russian Academy of Sciences, St-Petersburg, Russia; 3 Service of Nephrology, Centre Hospitalier Universitaire Vaudois, Lausanne, Switzerland; 4 Genomic Technologies Facility, University of Lausanne, Lausanne, Switzerland

**Keywords:** Glomerulus, Glomerulus

## Abstract

Glomerular filtration rate (GFR), or the rate of primary urine formation, is the key indicator of renal function. Studies have demonstrated that GFR exhibits significant circadian rhythmicity and, that these rhythms are disrupted in a number of pathologies. Here, we tested a hypothesis that the circadian rhythm of GFR is driven by intrinsic glomerular circadian clocks. We used mice lacking the circadian clock protein BMAL1 specifically in podocytes, highly specialized glomerular cells critically involved in the process of glomerular filtration (Bmal1^lox/lox^/Nphs2-rtTA/LC1 or, cKO mice). Circadian transcriptome profiling performed on isolated glomeruli from control and cKO mice revealed that the circadian clock controls expression of multiple genes encoding proteins essential for normal podocyte function. Direct assessment of glomerular filtration by inulin clearance demonstrated that circadian rhythmicity in GFR was lost in cKO mice that displayed an ultradian rhythm of GFR with 12-h periodicity. The disruption of circadian rhythmicity in GFR was paralleled by significant changes in circadian patterns of urinary creatinine, sodium, potassium and water excretion and by alteration in the diurnal pattern of plasma aldosterone levels. Collectively, these results indicate that the intrinsic circadian clock in podocytes participate in circadian rhythmicity of GFR.

## Introduction

The kidney plays a pivotal role in maintaining homeostasis. The homeostatic equilibrium is continuously challenged by a variety of external and internal factors, including those affected by circadian rhythms. Accordingly, it has long been recognized that homeostatic control over extracellular fluids requires continuous adjustment of diverse specific renal functions throughout the circadian cycle. For instance, renal blood flow (RBF), renal tissue oxygenation, renal cortico-medullary osmotic gradient, glomerular filtration rate (GFR) and tubular reabsorption/secretion of water and major electrolytes have been shown to display circadian rhythms with apparently similar kinetics characterized by a trough in the middle of the biological night and peak values in the middle of the active phase (reviewed in^1,2^). It has been hypothesized that these rhythms are driven, at least in part, by the circadian clock, a molecular timer formed by several transcriptional-translational feedback loops that oscillate with the periodicity of ~24 hours in a self-sustained and cell-autonomous manner. The core of the circadian clock imposes functional rhythms via the temporal control over transcription, translation and posttranslational modifications (reviewed in^3^).

Numerous studies have examined the role of the circadian clock in renal function. Analyses of mouse models with null mutations in core clock genes revealed that the circadian clock controls a large number of genes involved in diverse homeostatic renal functions^4,5^. Functionally, disruption of the circadian clock leads to dramatic changes in circadian patterns of urinary excretion of water and major electrolytes, impairment in cortico-medullary osmotic gradient and, loss of blood pressure (BP) control^6–9^. Recent research has addressed the role of *intrinsic* renal circadian clocks. Nikolaeva *et al*. have shown that conditional disruption of the essential circadian transcriptional activator Bmal1 in the renal tubule results in dysregulation of a wide range of intrarenal and systemic metabolic processes and, in impairment in renal xenobiotic elimination^5^. Tokonami *et al*. have demonstrated that ablation of the circadian clock specifically in renin-secreting granular cells resulted in a complex renal phenotype characterized by impaired handling of sodium, water, calcium and magnesium, a significantly modified circadian pattern of plasma aldosterone levels and, decreased BP^10^. Collectively, these studies have clearly established an important role for the circadian clock in renal tubular function.

The role of the circadian clock in the process of glomerular filtration remains much less understood. It has been shown that GFR display circadian oscillations with an amplitude of 20–40% when measured by inulin clearance^11–13^. The GFR depends on the difference between glomerular capillary and Bowman’s space hydrostatic pressure, the transcapillary oncotic pressure and the ultrafiltration coefficient (K_f_) which, in turn, depends on the hydraulic permeability of the glomerular filter and on the effective filtration surface. Studies have shown that circadian oscillations in GFR are independent of circadian rhythms in systemic blood pressure^14^ and, of the sympathetic renal innervation^15^. Moreover, Koopman *et al*., have shown that the GFR rhythm is not due to differences in posture and food intake over the circadian cycle^16^. Plasma albumin and total protein levels exhibit similar with GFR circadian patterns therefore acting as factors opposing GFR oscillations^17–19^. Altogether, these data raised the possibility that circadian rhythm in GFR is driven, at least in part, by intrinsic circadian clocks located in glomerular cells and/or juxtaglomerular apparatus. Here, we addressed the role of the circadian clock in podocytes, which are highly specialized epithelial cells that cover the outer surface of glomerular capillaries and that have been proposed to actively participate in the control of K_f_^20,21^.

## Results

### The *Bmal1* expression in podocytes

**Figure 1:**
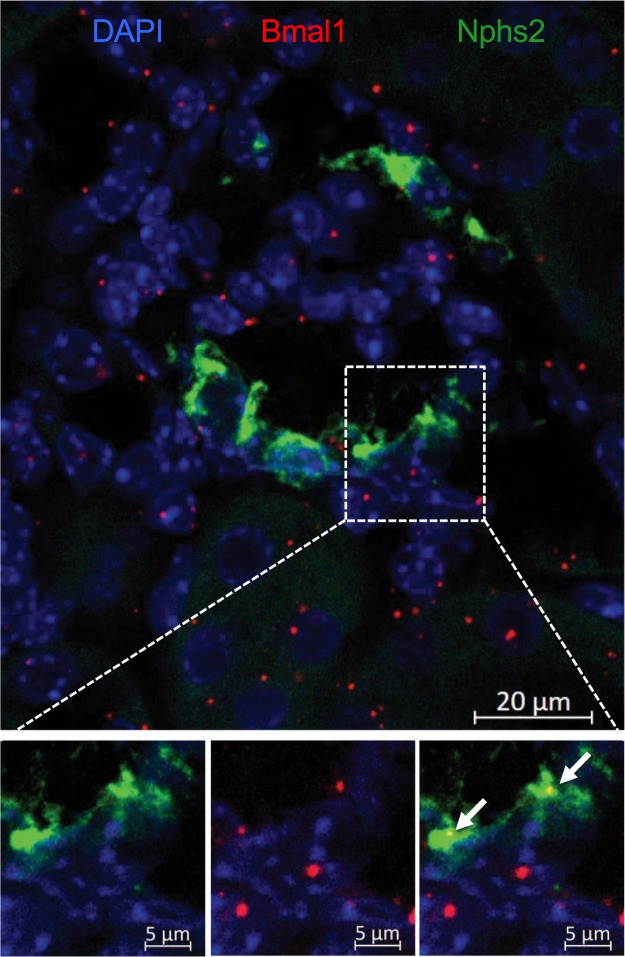
Bmal1 expression in podocytes. Representative RNAscope staining of Bmal1 (red) and Nphs2 (green) RNAs in a kidney section of Control mice. Area in the white rectangle is shown enlarged in the lower panel. White arrows indicate Bmal1 RNA molecules co-localized with Nphs2 staining.

Although it is generally assumed that the circadian clock machinery is present in nearly all eukaryotic cells, the expression and abundance of core clock components in podocytes has not been tested. To address this question, we performed RNAscope *in situ* hybridization of mouse kidneys with probes specific to Bmal1 and to Nphs2 (podocin, a critical element of the filtration slit specifically expressed in podocytes). As shown in Fig. 1, Bmal1 and Nphs2 mRNAs are co-localized within a subset of glomerular cells, thereby confirming the expression of Bmal1 in podocytes. The Bmal1 mRNA content measured by image quantitation revealed that 16.2 ± 2.4% (n = 14 glomeruli (SEM)) of glomerular Bmal1 mRNA molecules are expressed in podocytes, while podocytes represent ~30% of all glomerular cells, according to current estimates^22,23^. These results suggested that podocytes exhibit lower Bmal1 expression compared to the other glomerular cell types.

### The *Bmal1*^lox/lox^/Nphs2-rtTA/LC1 conditional knockout (cKO) model

To study the role of the circadian clock in podocytes we generated mice with podocyte-specific doxycycline (DOX)-inducible inactivation of the *Arntl* (*Bmal1*) gene encoding BMAL1, an indispensable transcriptional activator in the circadian clock machinery. To this aim, mice bearing floxed *Bmal1* alleles^24^ were crossed with *Nphs2*-rtTA/Cre transgenic mice commonly used for podocyte-specific conditional gene targeting^25^. Inactivation of the *Bmal1* gene was induced by 2-weeks treatment with DOX (2 mg/ml in drinking water) of 8-weeks old *Bmal1*^lox/lox^/Nphs2-rtTA/LC1 mice (hereafter referred to as conditional knockout mice or cKO mice). The same DOX treatment was provided to their littermate controls (*Bmal1*^lox/lox^ mice, hereafter referred to as Control mice). All experiments were performed 1 month after the end of DOX treatment in order to avoid potential side effects of DOX on renal function. The model was validated by demonstration of Cre-mediated genomic excision of floxed *Bmal1* alleles in the kidney and in isolated glomeruli from cKO mice (Supplementary Fig. 1A) and, by RT-PCR analysis performed on RNAs extracted from glomeruli isolated at different circadian time-points from kidneys of Control and cKO mice (Supplementary Fig. 1B). Body weights, 24-hour food and water intake (Supplementary Table 1) as well as physical activity rhythms (Supplementary Fig. 2) were not different between Control and cKO mice. The cKO mice did not show any obvious morphological and ultrastructural abnormalities and did not develop proteinuria (Supplementary Fig. 3).

### Transcriptome profiling of glomeruli isolated from Control and cKO mice

**Figure 2:**
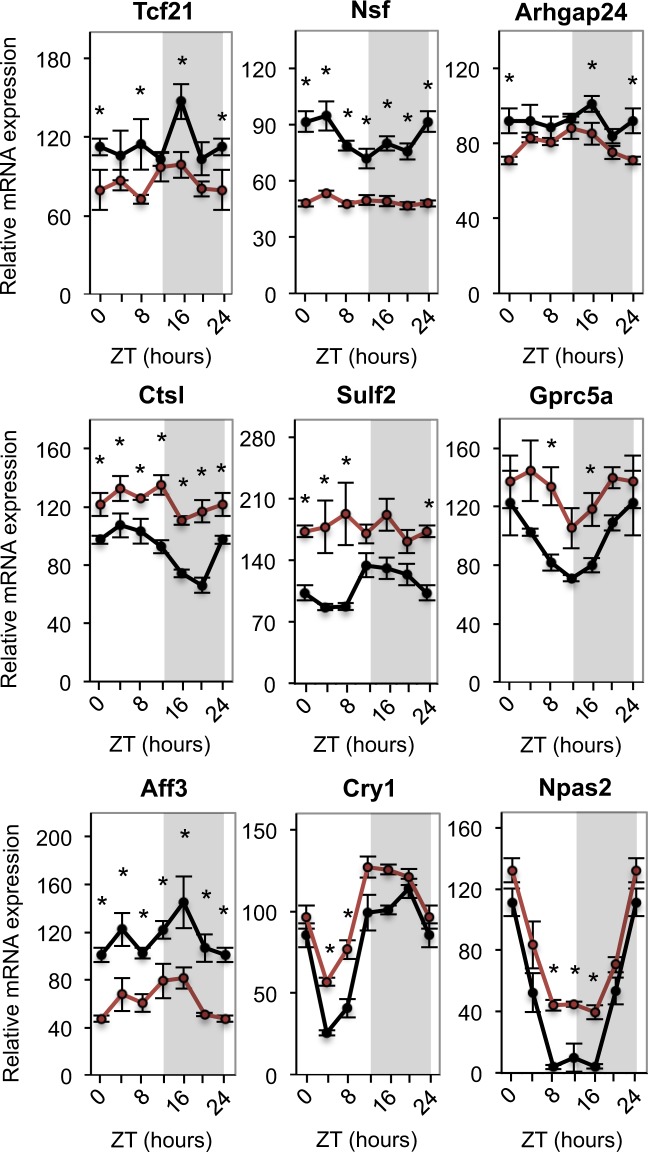
Analysis of glomeruli transcriptome revealed altered expression of genes involved in diverse podocyte-specific processes or being part of the circadian clock core. mRNA expression profiles of Tcf21, Nsf, Arhgap24, Ctsl, Sulf2, Gprc5a, Aff3, Cry1 and Npas2 in Control (black) and cKO (red) glomeruli. Values are means ± SEM. Statistical analyses were performed with the R package *limma*^48^. Contrasts between cKO and Control at each time point were combined into one F-test. *Time point with difference after post-hoc classification of significant genes (false discovery rate <5%). n = 6 mice/time-point/genotype.

To identify molecular pathways controlled by the circadian clock in podocytes we performed transcriptome profiling of glomeruli isolated from kidneys of Control and cKO mice at six different circadian time-points (ZT0, ZT4, ZT8, ZT12, ZT16 and ZT20; ZT – Zeitgeber or circadian time; ZT0 is the time of light-on and ZT12 is the time of light-off). Transcriptome analysis revealed multiple genes critically involved in a variety of podocyte-specific processes and exhibiting differential expression levels in glomeruli of Control and cKO mice (Fig. 2 and Supplementary Table 2). A significant reduction in expression levels (at one or more circadian time-points) was observed, e.g., for: Tcf21, a transcriptional factor playing a key role in podocyte differentiation and maintenance^26^; N-Ethylmaleimide Sensitive Factor (Nsf) and G Protein Subunit Alpha 12 (Gna12), genes involved in the organization and integrity of podocyte cytoskeleton^27,28^; Uncoupling protein 2 (UCP2) and Farnesoid X-Activated Receptor (FXR or Nr1h4), key proteins controlling renal metabolism^29,30^; and Rho GTPase Activating Protein 24 (Arhgap24), an enzyme critical for podocyte interaction with the basement membrane^31^. An increased expression was found, e.g., in: Cathepsin L (Ctsl), a protease highly expressed in the foot processes and important for normal podocyte architecture^32^, sulfatase 2 (Sulf2), an enzyme that controls paracrine interglomerular communication between podocytes and endothelial cells^33^ and Gprc5a, a highly podocyte-specific orphan G protein-coupled receptor which controls the thickness of the glomerular basement membrane^34^. Interestingly, among the circadian core clock genes, only Cry1, Npas2, Rora and Rorc displayed significant changes in the expression levels in glomeruli of cKO mice. The heterogeneous cellular composition of glomeruli and lower than the mean expression levels of Bmal1 in podocytes (see above) are possible explanations for these results.

### cKO mice lose circadian rhythmicity in GFR

**Figure 3:**
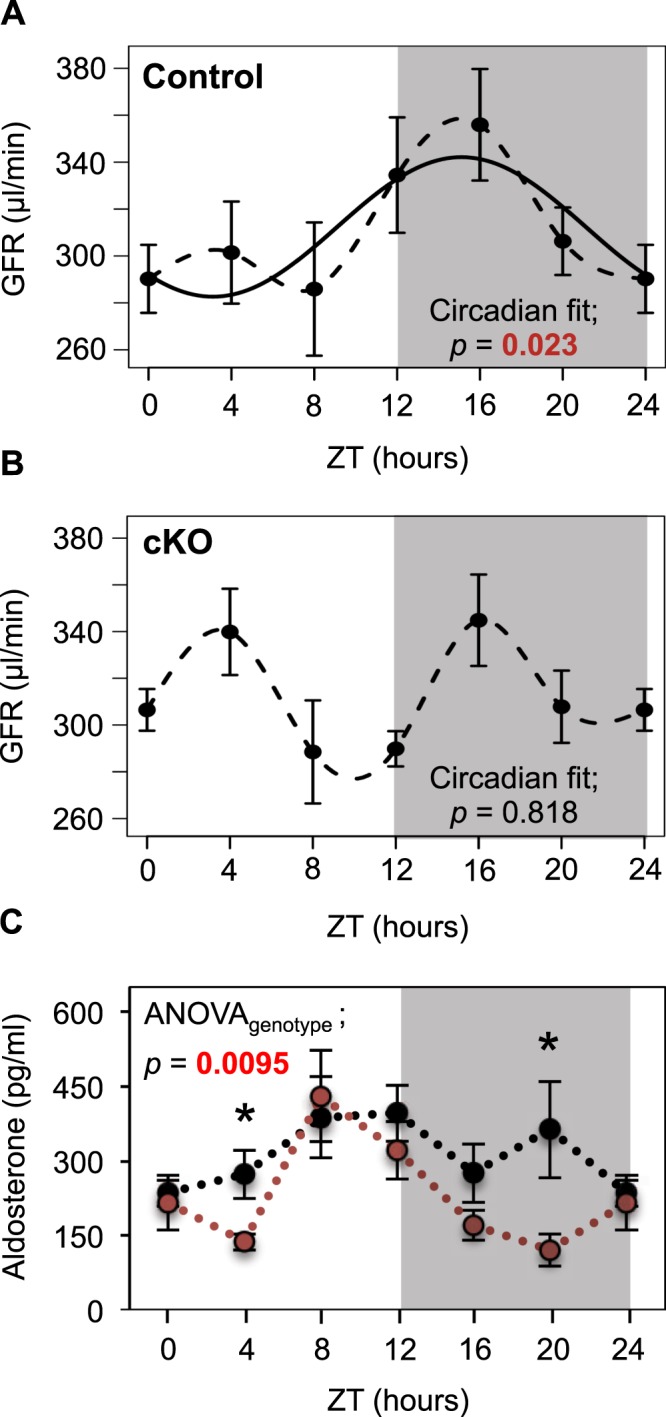
Circadian pattern of GFR is disrupted in cKO mice. Temporal profiles of GFR in (**A**) Control and (**B**) cKO mice. (**C**) Temporal profile of plasma aldosterone levels in Control (black) and cKO (red) mice. Values are means ± SEM. *p < 0.05 (unpaired *t* test). Circadian fit were analyzed with a linear model of a pair of cosine curves with a period of 24 hours. (**A**,**B**) Sin and Cos coefficients were combined into one contrast with the ‘glht’ (generalized linear hypothesis test) function of the R package ‘multcomp’^49^. Effect on aldosterone levels was tested with two-way Anova with genotypes and time points as factors. n = 6 mice/time-point/genotype, except n = 5 for cKO mice at ZT16.

**Figure 4:**
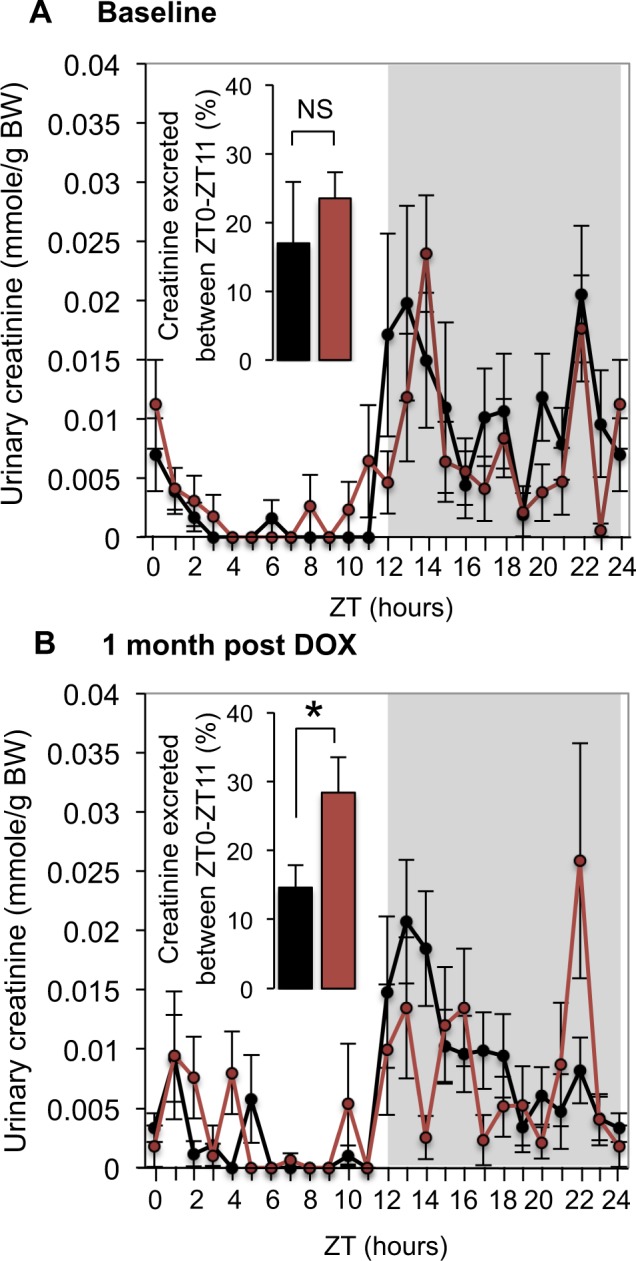
Urinary creatinine excretion during the inactive (light) phase (ZT0-ZT11) is higher in cKO mice. Profiles of every-hour urinary creatinine excretion in Control (black) and cKO (red) mice in (**A**) baseline (before DOX treatment) and (**B**) one month after the end of the DOX treatment. Bar plots represent the percentage of 24-hour urinary creatinine excretion excreted during the inactive (light) phase (ZT0-ZT11). *p < 0.05 (unpaired *t* test). n = 10 for cKO mice after DOX treatment, n = 11 in all other groups.

To determine whether the intrinsic circadian clock in podocytes participates in the control of GFR we compared circadian patterns of inulin clearance in Control and cKO mice. As shown in Fig. 3A, GFR in Control mice exhibited significant circadian rhythm (circadian fit p-value = 0.023) with an acrophase at ZT16 and a trough during the light (rest) phase. This circadian pattern in GFR was disrupted in cKO mice (circadian fit p-value = 0.336) that displayed an ultradian rhythm (12-hours period fit p-value = 0.003) with two peaks, the first at ZT4 and the second at ZT16 (Fig. 3B). Importantly, the integrated 24-hour GFR was not different between Control and cKO mice (p = 0.985, ANOVA). Because alterations in GFR are expected to elicit reciprocal adjustments in renin-angiotensin-aldosterone system (RAAS), which is a part of the tubulo-glomerular feedback (TGF) mechanism, we examined the circadian pattern of plasma aldosterone levels in Control and cKO mice. As shown in Fig. 3C, this pattern was strikingly altered in cKO mice (p = 0.0095, ANOVA). Of note, maximal values of GFR in cKO mice (ZT4 and ZT16) negatively correlated with minimal values in plasma aldosterone levels (ZT4 and ZT20), as could be expected from the general concept of TGF. As another independent approach to corroborate these results, we analyzed circadian profiles of urinary creatinine excretion in hourly collected urines. As shown in Fig. 4A, at baseline (before DOX treatment), both Control and cKO mice displayed similar patterns of urinary creatinine excretion with the percentage of creatinine excreted during the light phase (ZT0 to ZT11) not different between the two genotypes. One month after the end of DOX treatment, the percentage of creatinine excreted during the light phase was significantly higher in cKO mice compared to Control mice, thereby confirming the role of the circadian clock in podocytes in the control of GFR (Fig. 4B). A similar disruption of urinary excretory rhythms in cKO mice was observed for sodium, potassium and urine volume (Supplementary Fig. 4). However, the total 24-hour excretory rates for creatinine, sodium, potassium and water (Supplementary Table 1) as well as basic plasma parameters (Supplementary Table 1) and BP (Supplementary Fig. 5) were not different between Control and cKO mice.

## Discussion

The cellular origin and molecular mechanisms underlying the circadian rhythm of GFR have intrigued physiologists and clinicians for decades. Interest into this field was stimulated by clinical observations that the amplitude of circadian oscillations in GFR is reduced with age^35^ and, that the circadian rhythm in GFR is dampened or inverted in patients with the nephrotic syndrome^14^, heart failure^36^ or cirrhosis^12^. Early studies demonstrating that the circadian rhythm in GFR is independent on circadian oscillations in BP, cardiac output and sympathetic regulation suggested that an intrarenal self-autonomous mechanism(s) might be involved^14,15^. Here, we hypothesized that circadian oscillations in GFR are driven, at least in part, by the intrinsic circadian clock in podocytes, the highly specialized terminally differentiated glomerular cells that are essential for the formation of the glomerular filtration barrier and that are actively involved in control of GFR by regulating slit diaphragm and by influencing properties of glomerular endothelial cells via paracrine factors^20,21^. This hypothesis was tested at the transcriptional and functional levels.

Current estimates suggest that 10 to 50% of all cellular transcripts are rhythmic^37^. It has been shown that a majority of oscillating transcripts (with the exception of transcripts encoding proteins of the core clock machinery) exhibit tissue-specific expression patterns and/or display tissue-specific oscillation amplitudes and phases^37^. Accordingly, it has been proposed that the main role of peripheral circadian clocks consists in the dynamic adjustment of tissue-specific physiological functions. Recent studies demonstrating that the kidney exhibits one of the highest levels of circadian transcripts across mammalian tissues have underscored the potential importance of circadian clocks in renal cells^37^. Here, we showed that inactivation of the intrinsic circadian clock in podocytes led to significant alterations in expression patterns of multiple genes that have been implicated in diverse cellular processes critical for podocyte function, including podocyte differentiation, metabolism, cytoskeleton organization and adhesion. Polymorphic variants in several genes have been associated with human glomerular disease. For instance, single nucleotide polymorphisms (SNPs) in Ucp2 have been linked to decreased GFR in type 1 diabetic patients (T1D)^38^, SNPs in the Arhgap24 gene are associated with the focal segmental glomerulosclerosis (FSGS)^31,39^, SNPs in the AF4/FMR2 Family Member 3 (Aff3) gene are associated with diabetic end stage renal disease (ESRD) in T1D patients^40^ and, Ctsl levels show strong positive correlation with proteinuria in chronic kidney disease (CKD)^32^. Collectively, these results suggested that the intrinsic circadian clock is significantly involved in the control of podocyte transcriptome and that transcriptional changes in cKO mice may result in functional alterations.

We further provided three lines of functional evidence demonstrating that the intrinsic circadian clock in podocytes plays an essential role in the maintenance of the circadian rhythm of GFR. First, disruption of circadian rhythms of GFR in cKO mice was revealed through the direct assessment of the circadian pattern of GFR using inulin clearance. Second, inactivation of the circadian clock in podocytes was shown to increase the rate of urinary creatinine excretion in cKO mice during the inactive phase. Third, loss in circadian rhythmicity of GFR was paralleled by alterations in the circadian pattern of plasma aldosterone levels. Importantly, the circadian clock deficiency did not result in proteinuria and did not affect the integrated 24-hour GFR. This indicates that the main role of the circadian clock in podocytes consists in the maintenance of the circadian rhythm in GFR and not, or to a lesser extent, in the control of other specific cellular functions in podocyte (at least in unstressed conditions). These results, together with evidence from another recent study that addressed the role of intrinsic circadian clocks in the renal tubule^5^, support the idea that circadian clocks serve very specialized renal functions. Indeed, Nikolaeva *et al*. have shown that conditional ablation of the circadian clock in tubular cells resulted in impaired secretion of organic anions, but tubular handling of water and major electrolytes as well as the glomerular function were fully preserved in this model^5^. Our study also provides additional evidence for the intrinsic glomerular origin of circadian oscillations in GFR since circadian rhythm in BP was fully maintained in cKO mice. Collectively, these results demonstrate that intrinsic renal circadian clocks drive circadian oscillations in a number of essential renal functions, including glomerular filtration.

The cause(s) of disruption of circadian rhythms in GFR in patients with cardiovascular, renal or liver diseases remains unknown. However, it is well established that intrinsic circadian clocks in peripheral tissues are orchestrated by external circadian cues, of which systemic circulating factors (hormones, food, food metabolites) are probably most important for renal cells. Accordingly one may hypothesize that profound changes in the circulating metabolome that are characteristic for the aforementioned disorders may cause dysfunction in circadian clocks within podocytes and/or other glomerular cells.

## Methods

### Animals

The procedures used to generate, and the characterization of, *Bmal1*^*lox/lox*^ and *LC-1 Cre* mice were described previously^41^. The *Nphs2*^*lox/*+^ mice were obtained from The Jackson Laboratory. The three mouse lines used in this study are inbred strains, bred on the genetic background of *C57BL*/6J mice. The animals were maintained *ad libitum* on the standard laboratory chow diet (KLIBA NAFAG diet 3800). Before all experiments, mice were adapted to a 12-hour light/ 12 hour dark cycle for two weeks. All experiments were performed on male mice.

### PCR for recombined *Bmal1* allele

Excision of floxed *Bmal1* allele was assessed on cDNA or gDNA using the following primers; F_5′-TGG ACA CAG ACA AAG ATG ACC CTC A-3′ and R_5′-TCC CTC GGT CAC ATC CTA CGA CA-3′ (for cDNA) and F_5′-AGG GAC AGG CCA AAA GTC TG-3′ and R_5′-GGC ACA TGT CTT AAT CTA CCC-3′ (for gDNA).

### Metabolic cages

Mice were individually housed in metabolic cages (Tecniplast) and subjected to 3 days of adaptation before urine collection. The every hour urine collection was performed using a 12-chanel peristaltic pump, as previously described by Nikolaeva *et al*.^8^.

### GFR

GFR measurements were performed on anesthetized animals with inulin-FITC as previously described^42^. Briefly, ~2.5% FITC-inulin (2 μl/g BW) was injected into the retro-orbital plexus of Control or cKO mice. Blood collection was performed 3, 7, 10, 15, 40 and 60 minutes post-injection and inulin clearance was calculated using a two phase exponential decay curve model^43^. A total of 36 Control mice and 36 cKO mice were used for GFR measurement (6 mice/time-point/genotype).

### Glomeruli isolation and RNA extraction

Isolation of mouse glomeruli was performed as previously described by Takemoto *et al*.^44^. Briefly, mice were anesthetized by intraperitoneal injection of Ketamine:Xylazine (1:1) and Dynabeads M-450 Tosylactivated (Invitrogen) perfused through the abdominal aorta. After perfusion, the 2 kidneys were harvested and minced into small pieces, before to be digested with collagenase at 30 °C for 30 minutes. The digested tissue was filtered and cell suspension centrifuged. Dynabeads containing-glomeruli were separated with a magnetic particle concentrator and washed. This method allows to recover ~10,000 glomeruli per kidney. Representative image of isolated glomeruli is shown in Supplementary Fig. 6. RNA from isolated glomeruli was directly extracted using RNAeasy MiniElute Spin Column (Qiagen). and 200 ng of purified RNA were used for RNA sequencing. A total of 36 Control mice and 36 cKO mice were used for the transcriptome experiments (6 mice/time-point/genotype).

### Plasma aldosterone

Heparinized plasma was collected from anesthetized mouse at different ZT and aldosterone level measured by radioimmunoassay (DPC).

### Arterial blood pressure

Blood pressure was measured with telemetry system (DSI) in freely moving mice.

### Locomotor activity

The circadian patterns of general locomotor activity were measured by using the Mouse-E-Motion system (Infra-E-Motion Gmbh). This system allows real-time measurements of general motion activity in mice housed in normal laboratory cages.

### RNA scope

The RNAscope analysis was performed according *to the* manufacturer’s protocol on kidneys harvested at ZT4.

### RNA-seq

RNA-seq libraries were prepared using 200 ng of total RNA as described in Nikolaeva *et al*.^5^. Illumina TruSeq SR Cluster Kit v4 reagents were used. Sequencing data were processed as described in^45^ using *Mus musculus*.*GRCm38*.86 gene annotation. Statistical analysis was performed in R (version 3.4.0). Genes with low counts were filtered out according to the rule of 1 count per million (cpm) in at least 1 sample. Library sizes were scaled using TMM normalization and log-transformed into counts per million (CPM) using voom^46^.

Principal component analysis showed a large variability between replicate samples. Two factors of unwanted variation were removed using the RUVs function (R package RUVSeq v. 1.10.0^47^). Differential expression between knock-out and wild-type was computed using limma^48^. A linear model with a factor for each combination of time point and genotyope was used. Factors to correct for batch effect and unwanted variation were also added in the design matrix. Differences in gene expression levels between KO and WT animals at each time points were combined into one F-test. Genes with a false discovery rate <5% were considered significant. The limma function ‘classifyTestsF’ was used to classify time point as significant or not for the selected genes.

### Statistics

All data are expressed as mean ± SEM. Statistical differences between two groups were determined by Student’s 2-tailed *t* test. Differences between groups containing 2 variables were assessed by 2-way ANOVA. For repeated measurements, 2-way ANOVA was used. *P* < 0.05 was considered significant.

### Study approval

All experiments with animals were performed in accordance with the Swiss guidelines for animal care, which conform to the National Institutes of Health animal care guidelines. All animal protocols were approved by Swiss veterinary authorities (authorisation #27750).

## Supplementary information

Supplementary information
