# Supplementary material for: The intrinsic circadian clock in podocytes controls glomerular filtration rate
Source: Sci Rep. 2019 Nov 6;9:16089. doi: 10.1038/s41598-019-52682-9 (PMC6838779; doi:10.1038/s41598-019-52682-9)
Supplement: Supplementary file 1 — Supplementary information [file 41598_2019_52682_MOESM1_ESM.pdf]

SUPPLEMENTARY INFORMATION

The intrinsic circadian clock in podocytes controls glomerular filtration rate.

Camille Ansermet[1#], Gabriel Centeno[1#], Svetlana Nikolaeva[1,2], Marc P. Maillard[3], Sylvain Pradervand[4] and Dmitri Firsov[1*]

[1] - Department of Pharmacology and Toxicology, University of Lausanne, Switzerland

[2] - Institute of Evolutionary Physiology and Biochemistry, Russian Academy of Sciences, St-Petersburg, Russia

[3] - Service of Nephrology, Centre Hospitalier Universitaire Vaudois, Lausanne, Switzerland

[4] - Genomic Technologies Facility, University of Lausanne, Switzerland

[#] - these authors contributed equally to this work

* - to whom correspondence should be addressed:

Dmitri Firsov, Department of Pharmacology and Toxicology, University of Lausanne, 27 rue du Bugnon, 1005 Lausanne, Switzerland. Phone: ++ 41-216925406

e-mail: dmitri.firsov@unil.ch

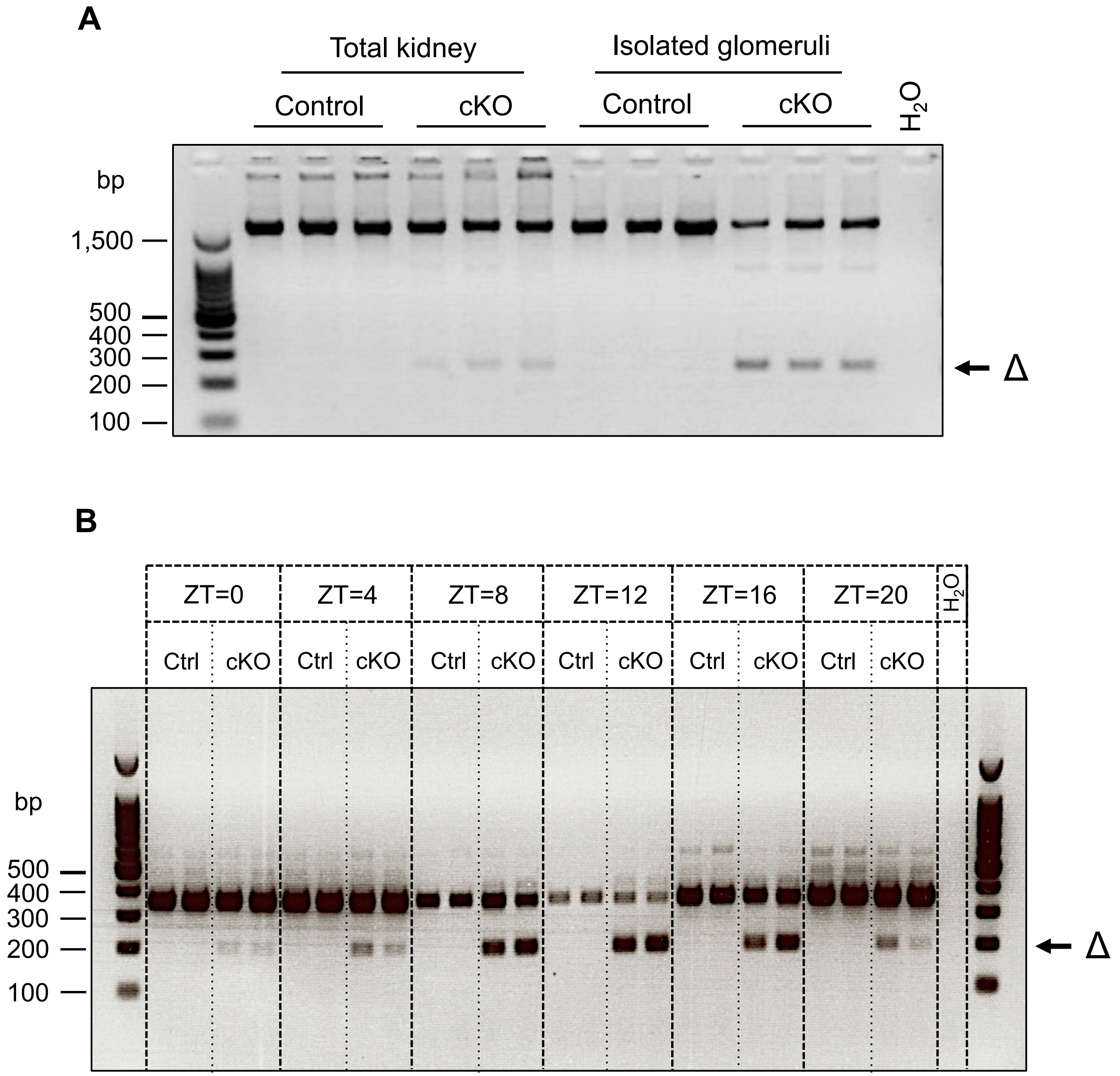

Supplementary Figure 1. (A) Genomic DNA *Bmal1* excision in Control and cKO total kidney extract and isolated glomeruli. **(B)** cDNA Bmal1 excision at different circadian timepoints in Control and cKO isolated glomeruli. Δ=excised Bmal1 fragment.

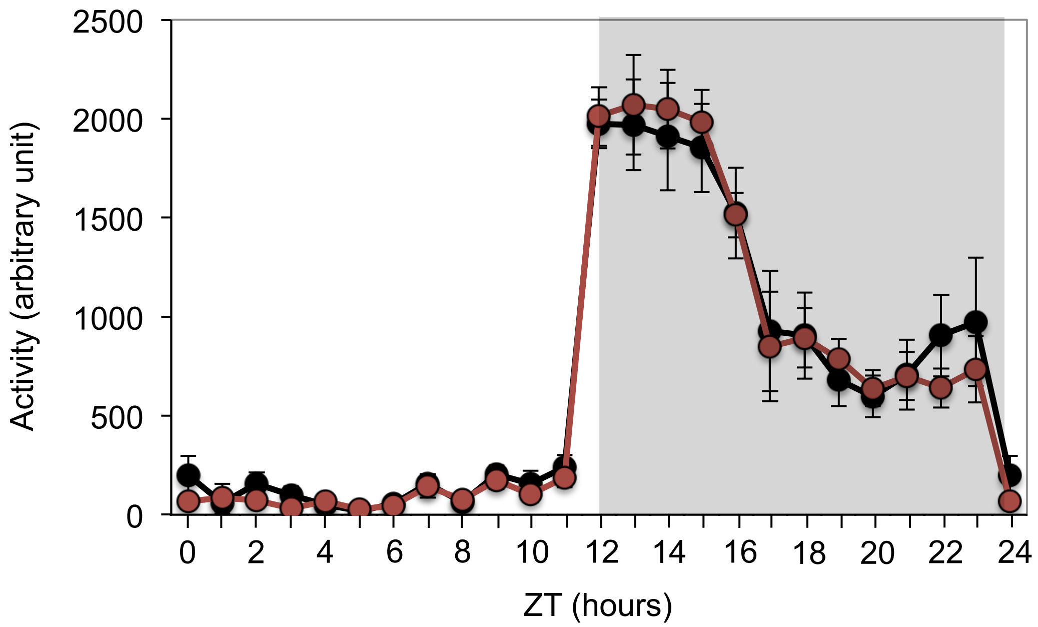

Supplementary Figure 2. Every-hour locomotor activity recorded in Control (black) and cKO (red) mice during 5 consecutive days. Values are means ± SEM. n=6 Control mice, n=6 cKO mice.

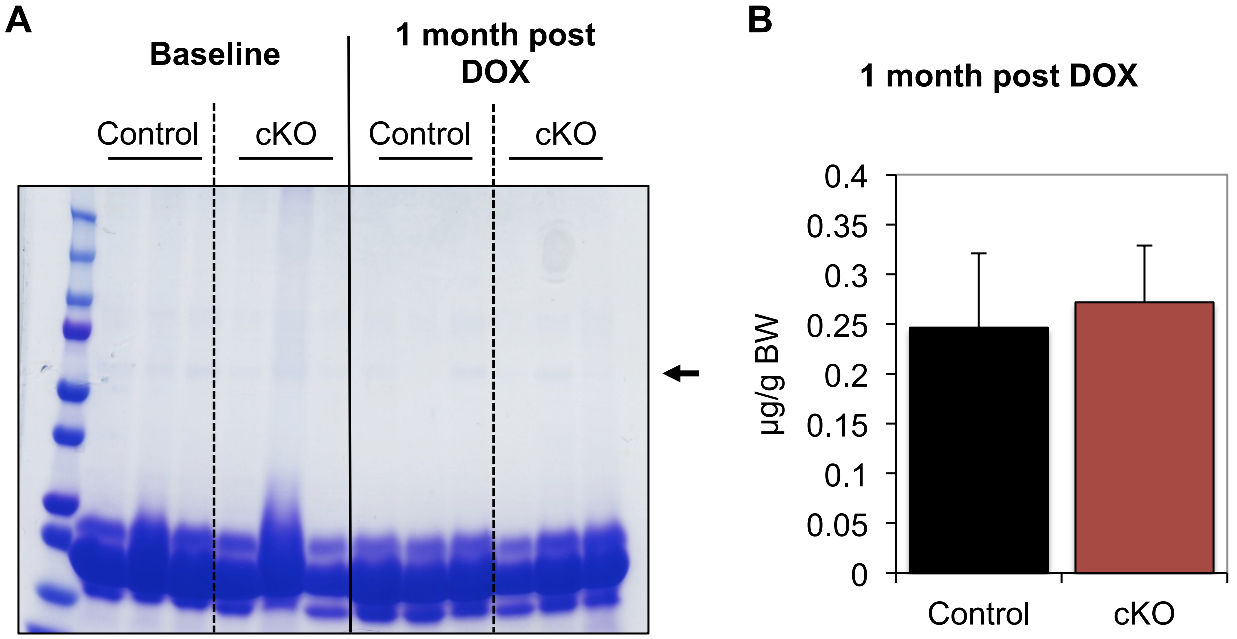

Supplementary Figure 3. (A) Coomassie blue-stained 24-hour urine of Control and cKO mice before (baseline) and 1 month after the DOX treatment. The arrow indicates the expected size for albumin (~65kDa). **(B)** Quantitative dosage of albumin in 24-hour urine collected 1 month after the DOX treatment.

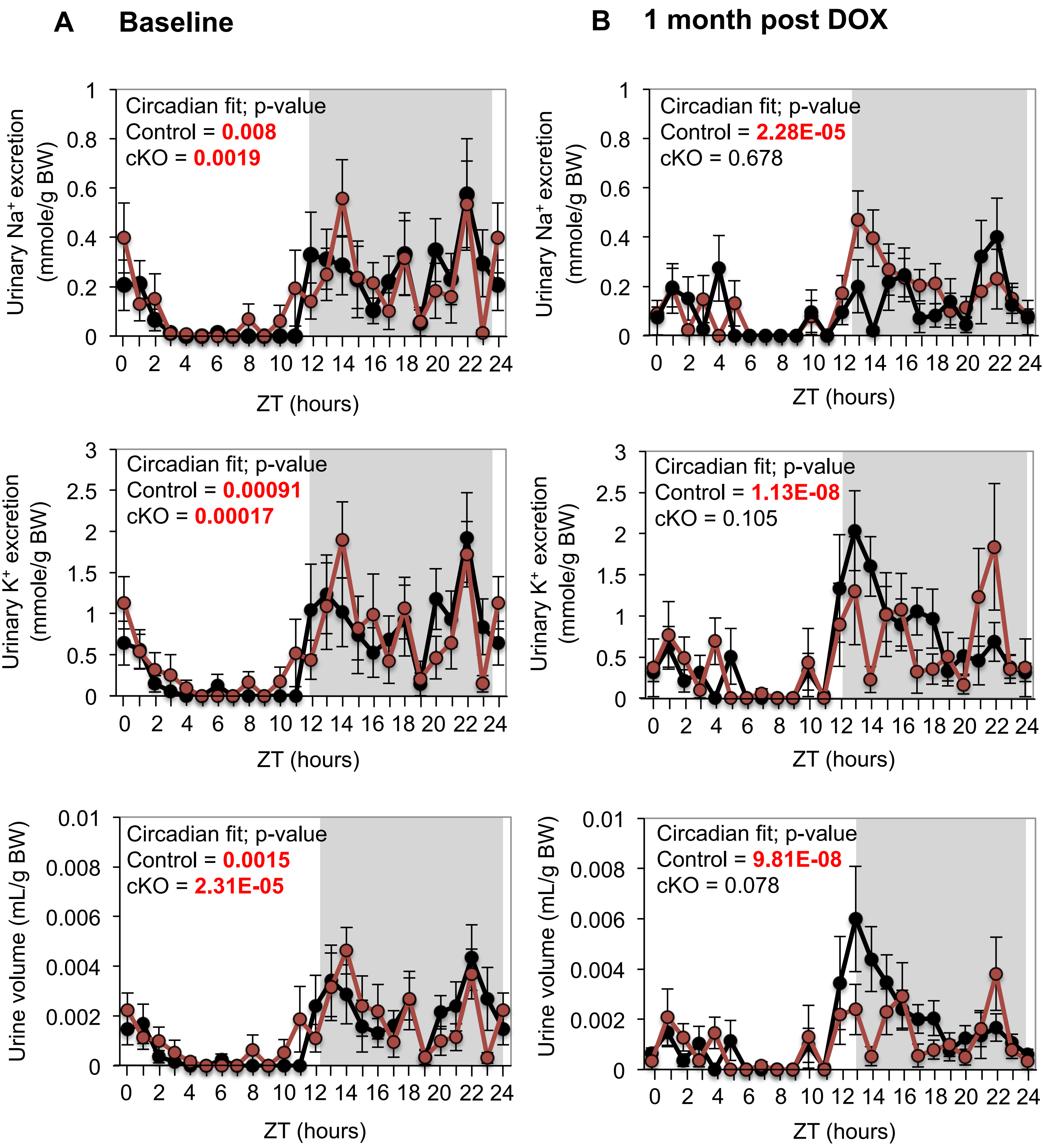

Supplementary Figure 4. Every-hour urinary Na^+, K^+ and water excretion of Control (black) and cKO (red) mice **(A)** before (baseline) and **(B)** 1 month after DOX treatment. Values are means ± SEM. Circadian fit were assessed with cosine curve fitting analysis. n=12 Control mice, n=11 cKO mice.

A Baseline

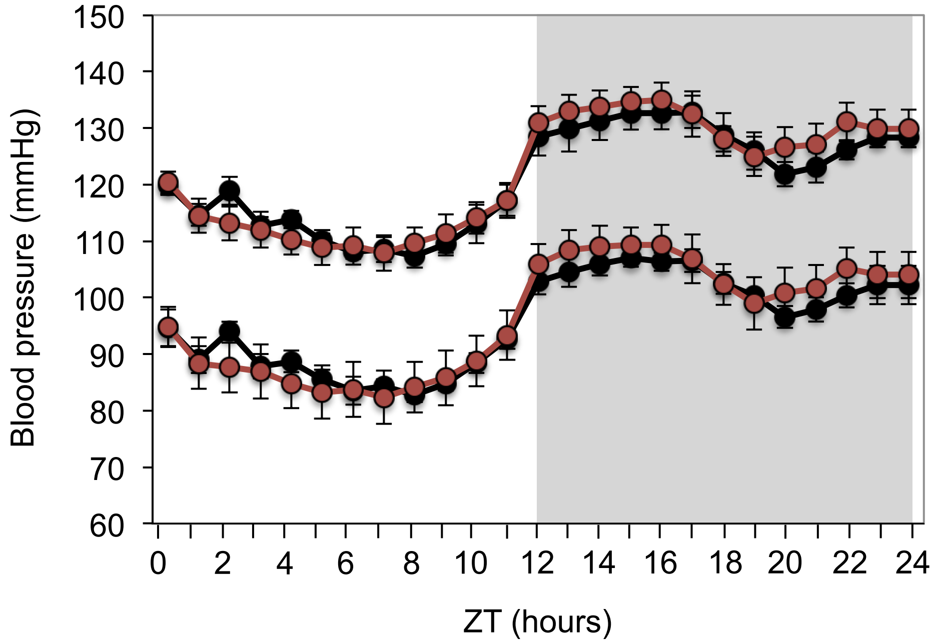

B 1 month post DOX

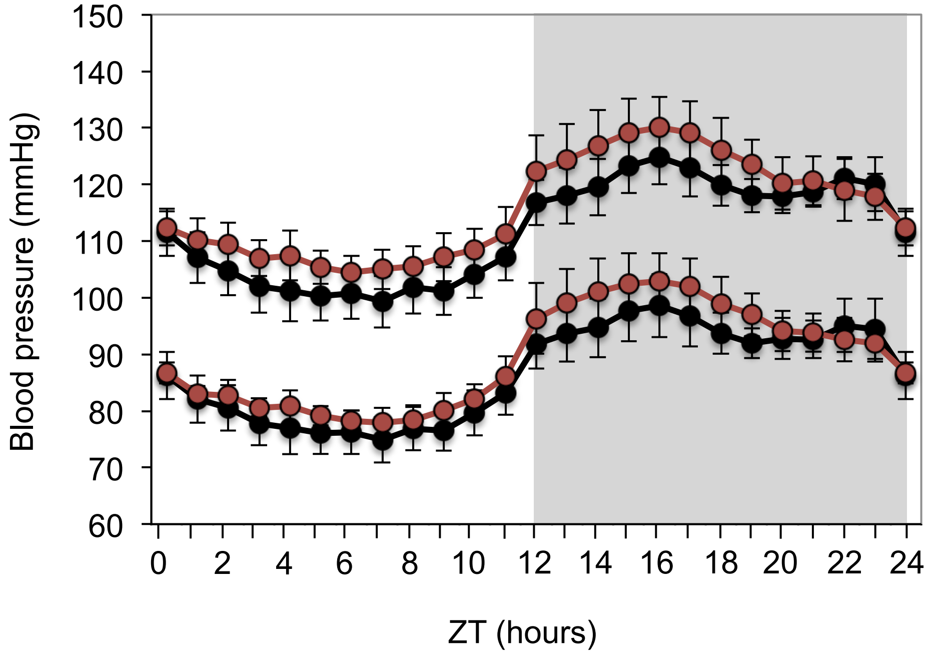

Supplementary Figure 5. Telemetry-measured systolic and diastolic blood pressure of Control (black) and cKO (red) mice **(A)** before (baseline) and **(B)** 1 month after DOX treatment. Each point corresponds to the mean ± SEM of 7 consecutive days of measurements. n=5 Control mice, n=5 cKO mice.

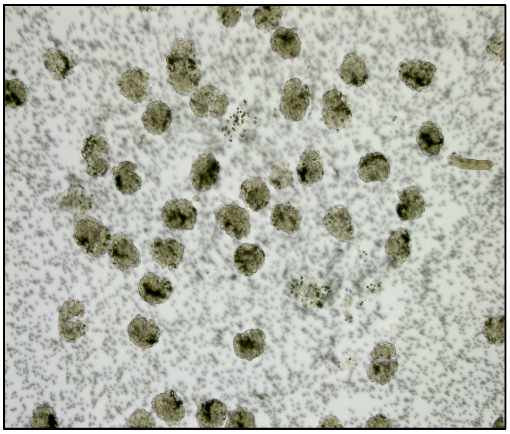

Supplementary Figure 6. Representative microscopic image of glomeruli recovered after isolation and washing steps and used for RNA sequencing. Magnification 100X. Bright field.

Supplementary Table 1. General parameters, 1 month post DOX.

	Control (n=6)	cKO (n=6)	p
Body weight (g)	28.16±0.43	27.95±0.63	NS
Food intake/g BW (g/g)	0.17±0.01	0.17±0.005	NS
Water intake/g BW (ml/g)	0.19±0.02	0.18±0.01	NS

<u>24-hour urine</u>

	Control (n=6)	cKO (n=6)	p
Urine volume/BW (ml/g)	0.029±0.005	0.016±0.004	NS
UVxNa+/g BW (µmol/g)	4.85±0.98	4.76±1.66	NS
UVxK+/g BW (µmol/g)	18.41±3.24	11.96±2.77	NS
Na+/K+	0.36±0.11	0.39±0.06	NS
UVxCreatinine/g BW (µmol/g)	0.19±0.03	0.16±0.03	NS

<u>Plasma (ZT6)</u>

	Control (n=6)	cKO (n=6)	p
Osmolality (mOsm/kg H2O)	320.6±1.1	323±2.9	NS
Na+ (mM)	154.6±0.7	153.5±0.5	NS
K+ (mM)	3.61±0.07	4.03±0.18	0.04
Creatinine (µM)	17.06±0.93	17.95±2.08	NS

Supplementary Table 2. Genes differentially expressed between Control and cKO glomeruli at one or more ZT

Gene	logFC_ZT0	logFC_ZT4	logFC_ZT8	logFC_ZT12	logFC_ZT16	logFC_ZT20	F	adj.P.Val	ZT (Posthoc test)
Nsf	-0.88	-0.80	-0.79	-0.48	-0.80	-0.63	76.12	5.1E-10	ZT0 ZT4 ZT8 ZT12 ZT16 ZT20
Adam11	2.51	3.29	2.72	1.90	2.72	2.90	57.89	5.1E-09	ZT0 ZT4 ZT8 ZT12 ZT16 ZT20
Ccdc43	0.81	0.89	0.60	0.34	0.54	0.61	47.41	2.9E-08	ZT0 ZT4 ZT8 ZT12 ZT16 ZT20
Fzd2	0.75	1.00	0.34	0.39	0.23	0.89	34.21	6.8E-07	ZT0 ZT4 ZT8 ZT12 ZT20
Aff3	-1.12	-0.96	-0.98	-0.61	-1.08	-0.81	30.52	1.8E-06	ZT0 ZT4 ZT8 ZT12 ZT16 ZT20
Psmc3ip	2.07	1.87	1.40	0.97	1.53	2.10	26.81	5.4E-06	ZT0 ZT4 ZT8 ZT12 ZT16 ZT20
Wnt8b	1.02	1.45	1.29	1.35	1.75	1.08	26.14	6.0E-06	ZT0 ZT4 ZT8 ZT12 ZT16 ZT20
Tlr7	-1.02	-0.96	-1.15	-0.56	-1.14	-0.89	25.39	7.0E-06	ZT0 ZT4 ZT8 ZT12 ZT16 ZT20
Ctsl	0.30	0.33	0.37	0.50	0.67	0.73	23.89	1.1E-05	ZT0 ZT4 ZT8 ZT12 ZT16 ZT20
Abca9	-0.92	-0.57	-0.60	-0.46	-0.65	-0.48	21.00	3.6E-05	ZT0 ZT4 ZT8 ZT12 ZT16 ZT20
Dpy19l3	0.22	0.05	0.07	0.60	0.41	0.29	20.55	4.0E-05	ZT12 ZT16 ZT20
Kpna2	-0.78	-0.79	-0.87	-0.42	-0.63	-0.98	20.20	4.3E-05	ZT0 ZT4 ZT8 ZT16 ZT20
Cry1	0.20	1.17	0.88	0.41	0.26	0.12	19.89	4.6E-05	ZT4 ZT8
2610204G07Rik	-2.19	-1.52	-1.17	-1.09	-1.77	-1.48	18.24	9.6E-05	ZT0 ZT4 ZT8 ZT12 ZT16 ZT20
Ablim3	0.41	0.40	0.69	0.72	0.99	0.77	17.42	1.4E-04	ZT8 ZT12 ZT16 ZT20
Rorc	0.05	1.12	0.68	0.13	-0.20	-0.03	16.80	1.8E-04	ZT4 ZT8
F3	-0.63	-0.14	-0.38	-0.39	-0.62	-0.42	16.10	2.4E-04	ZT0 ZT8 ZT12 ZT16 ZT20
Csdc2	0.23	0.70	1.26	1.67	1.40	0.48	16.09	2.4E-04	ZT8 ZT12 ZT16
Daam2	0.06	0.07	0.48	0.58	0.62	0.41	15.59	3.0E-04	ZT8 ZT12 ZT16 ZT20
Vmn1r32	4.61	3.35	3.89	4.03	2.44	5.05	14.02	7.0E-04	ZT0 ZT4 ZT8 ZT12 ZT20
Sulf2	0.83	1.01	1.07	0.39	0.52	0.43	14.01	7.0E-04	ZT0 ZT4 ZT8
Il34	-0.34	-0.20	-0.33	-0.09	-0.42	-0.29	13.75	7.9E-04	ZT0 ZT8 ZT16 ZT20
Thsd7a	-0.67	-0.12	-0.44	0.00	-0.17	-0.43	13.69	7.9E-04	ZT0 ZT8 ZT20
Gm12586	4.13	6.40	3.54	2.67	3.82	0.06	13.26	9.9E-04	ZT0 ZT4 ZT8 ZT16
Sybu	-0.48	-0.73	-0.40	-0.24	-0.31	-0.54	13.13	1.0E-03	ZT0 ZT4 ZT8 ZT20

Ggt5	0.05	0.19	0.37	0.70	1.06	0.26	13.04	1.0E-03	ZT12 ZT16
Gna12	-0.13	-0.13	-0.36	-0.25	-0.28	-0.10	13.02	1.0E-03	ZT8 ZT12 ZT16
Gaa	-0.39	-0.32	-0.34	-0.19	-0.28	-0.02	12.28	1.6E-03	ZT0 ZT4 ZT8 ZT16
Olfr1034	0.69	1.91	0.42	1.29	0.63	-0.12	12.09	1.8E-03	ZT4 ZT12
Npas2	0.18	0.59	3.62	4.24	3.24	0.60	11.50	2.7E-03	ZT8 ZT12 ZT16
Foxs1	0.18	0.53	1.98	1.88	0.71	0.19	11.42	2.7E-03	ZT8 ZT12
Nr1h4	0.65	-0.65	-0.29	0.47	-0.03	-0.36	11.41	2.7E-03	ZT0 ZT4 ZT12
Dusp15	0.13	0.59	0.89	1.05	0.74	0.00	11.33	2.7E-03	ZT8 ZT12 ZT16
Tcf21	-0.68	-0.28	-0.65	-0.14	-0.63	-0.31	11.26	2.8E-03	ZT0 ZT8 ZT16
Kcna6	-1.91	-0.57	-1.36	-0.90	-1.24	-1.30	11.15	2.9E-03	ZT0 ZT8 ZT16 ZT20
Nbl1	0.35	0.56	0.33	0.34	0.49	0.43	11.07	3.0E-03	ZT0 ZT4 ZT12 ZT16 ZT20
Ccbe1	-1.72	-1.17	-0.97	-0.82	-1.17	-0.56	10.97	3.2E-03	ZT0 ZT4 ZT16
Mgat5b	-1.36	-1.46	-0.74	-0.41	-0.81	-0.87	10.67	3.9E-03	ZT0 ZT4
Uaca	-0.30	-0.11	-0.29	-0.24	-0.31	-0.09	10.37	4.8E-03	ZT0 ZT8 ZT12 ZT16
Celf3	0.44	1.30	2.29	2.19	1.20	0.96	10.19	5.3E-03	ZT8 ZT12
Slc38a4	-0.36	-0.41	-0.43	-0.01	-0.54	-0.35	10.11	5.6E-03	ZT0 ZT4 ZT8 ZT16
Pmepa1	-0.33	-0.14	-0.37	-0.18	-0.49	-0.35	9.91	6.3E-03	ZT0 ZT8 ZT16 ZT20
sept.06	-0.32	-0.37	-0.46	-0.23	-0.63	-0.25	9.85	6.5E-03	ZT8 ZT16
1700052K11Rik	0.57	0.50	0.48	0.44	0.33	0.41	9.72	7.1E-03	ZT0 ZT4 ZT8 ZT12
Srrm4	-0.12	0.21	0.46	0.78	0.26	0.10	9.52	8.1E-03	ZT12
Creb3l1	-0.18	0.15	1.09	1.06	0.43	0.05	9.31	9.4E-03	ZT8 ZT12
Lpcat4	0.32	0.27	0.00	0.22	0.12	0.22	9.16	1.0E-02	ZT0 ZT4
Sowahd	-0.81	-0.37	-0.87	0.02	-0.66	-0.06	8.97	1.2E-02	ZT0 ZT8
Pcsk2	-0.10	-0.51	0.65	2.48	2.73	0.80	8.97	1.2E-02	ZT12 ZT16
Vgll3	-0.65	-0.37	-0.41	-0.34	-0.40	-0.87	8.86	1.3E-02	ZT0 ZT20
Fhod3	-0.50	-0.23	-0.12	-0.16	-0.48	0.10	8.80	1.3E-02	ZT0 ZT16
Prdx6	-0.10	-0.06	-0.16	-0.17	-0.37	-0.19	8.62	1.5E-02	ZT16
Kcnj2	0.65	0.65	0.82	0.32	0.41	0.66	8.41	1.8E-02	ZT0 ZT4 ZT8 ZT20
Ucp2	-0.44	-0.02	-0.29	-0.07	-0.49	-0.02	8.23	2.1E-02	ZT0 ZT16

Racgap1	-0.51	0.25	-0.52	-0.26	-0.05	-0.58	8.14	2.2E-02	ZT0 ZT8 ZT20
Gprc5a	0.21	0.42	0.78	0.53	0.64	0.34	8.08	2.2E-02	ZT8 ZT16
Ptchd4	-0.34	-0.49	-0.92	-0.53	-0.98	-0.70	8.05	2.3E-02	ZT8 ZT16
Wnt4	-0.75	-0.53	-0.36	-0.29	-0.54	-0.36	8.01	2.3E-02	ZT0
Arhgap24	-0.34	-0.10	-0.21	-0.05	-0.35	-0.12	7.86	2.6E-02	ZT0 ZT16
Gm4875	3.85	5.35	2.30	5.23	0.56	6.00	7.81	2.7E-02	ZT4 ZT12 ZT20
Gm12829	-1.59	-0.82	-1.13	-0.25	-0.41	-0.22	7.78	2.7E-02	ZT0
Hip1r	-0.03	0.09	0.34	0.52	0.16	0.13	7.72	2.8E-02	ZT12
Tspan11	1.10	1.49	1.11	1.67	1.42	1.36	7.52	3.4E-02	ZT4 ZT12
RP23-365O13.3	-0.80	0.00	-0.97	-0.41	-0.83	-0.52	7.50	3.4E-02	ZT8
Tmtc1	-0.43	-0.27	-0.25	-0.16	-0.49	-0.31	7.38	3.7E-02	ZT16
Dusp14	0.54	0.49	0.12	0.53	-0.18	0.06	7.36	3.7E-02	ZT0 ZT12
Rora	-0.06	-0.13	-0.14	-0.15	-0.24	-0.27	7.32	3.8E-02	ZT20
Itgb4	0.18	-0.02	-0.42	-0.07	0.16	0.66	7.20	4.3E-02	ZT20
Cyp2s1	0.07	0.46	0.12	0.05	0.01	0.73	7.05	4.8E-02	ZT20
Ccnjl	0.22	0.27	1.31	1.12	0.33	0.39	7.02	4.9E-02	ZT8
Leo1	-0.02	0.13	0.58	0.81	0.25	0.06	7.00	4.9E-02	ZT12
